# Plassembler: an automated bacterial plasmid assembly tool

**DOI:** 10.1093/bioinformatics/btad409

**Published:** 2023-06-27

**Authors:** George Bouras, Anna E Sheppard, Vijini Mallawaarachchi, Sarah Vreugde

**Affiliations:** Adelaide Medical School, Faculty of Health and Medical Sciences, The University of Adelaide, Adelaide, South Australia 5005, Australia; The Department of Surgery – Otolaryngology Head and Neck Surgery, Central Adelaide Local Health Network, Adelaide, South Australia 5000, Australia; School of Biological Sciences, The University of Adelaide, Adelaide, South Australia 5005, Australia; Flinders Accelerator for Microbiome Exploration, College of Science and Engineering, Flinders University, Bedford Park, Adelaide, South Australia 5042, Australia; Adelaide Medical School, Faculty of Health and Medical Sciences, The University of Adelaide, Adelaide, South Australia 5005, Australia; The Department of Surgery – Otolaryngology Head and Neck Surgery, Central Adelaide Local Health Network, Adelaide, South Australia 5000, Australia

## Abstract

**Summary:**

With recent advances in sequencing technologies, it is now possible to obtain near-perfect complete bacterial chromosome assemblies cheaply and efficiently by combining a long-read-first assembly approach with short-read polishing. However, existing methods for assembling bacterial plasmids from long-read-first assemblies often misassemble or even miss bacterial plasmids entirely and accordingly require manual curation. Plassembler was developed to provide a tool that automatically assembles and outputs bacterial plasmids using a hybrid assembly approach. It achieves increased accuracy and computational efficiency compared to the existing gold standard tool Unicycler by removing chromosomal reads from the input read sets using a mapping approach.

**Availability and implementation:**

Plassembler is implemented in Python and is installable as a bioconda package using ‘conda install -c bioconda plassembler’. The source code is available on GitHub at https://github.com/gbouras13/plassembler. The full benchmarking pipeline can be found at https://github.com/gbouras13/plassembler_simulation_benchmarking, while the benchmarking input FASTQ and output files can be found at https://doi.org/10.5281/zenodo.7996690.

## 1 Introduction

Advances in the accuracy of long-read sequencing have made near perfect bacterial genome assemblies attainable by combining long- and short-read sequencing technologies (Wick *et al.* 2023). Until recently, short-read-first hybrid assembly methods were favoured using tools such as Unicycler (Wick *et al.* 2017), which implements short read assembly using SPAdes (Bankevich *et al.* 2012). As long-read sequencing accuracy has continued to improve, the current best practice favours long-read-first assemblies supplemented with short-read polishing using tools such as Trycycler (Wick *et al.* 2021b), Dragonflye (https://github.com/rpetit3/dragonflye), or MicroPIPE (Murigneux *et al.* 2021).

A limitation of long-read-first assemblies is that small (<20 kb) plasmids are often missed by long read first assemblies, especially when ligation-based library preparation methods are used (Wick *et al.* 2021a). This may result in an incomplete picture of a sample’s plasmid mobilization and virulence potential, particularly for those with plasmids carrying antimicrobial resistance genes (Barry *et al.* 2019). In addition, long-read first assemblies often miss and misassemble small plasmids by doubling or tripling their length in assemblies (Wick and Holt 2019, Johnson *et al.* 2023), requiring manual intervention and curation. Accordingly, current best practice recommends hybrid short-read first assembly to recover small plasmids (Johnson *et al.* 2023, Wick *et al.* 2023). However, this method is computationally inefficient, as all input reads are assembled, including the majority that constitute the bacterial chromosome.

To improve computational efficiency, to increase accuracy, and to provide plasmid-only output that can be integrated with long-read-first pipelines chromosomal assemblies, we created Plassembler as a one-line tool that automatically outputs bacterial plasmid assemblies. Its increase in computational efficiency results from removing all reads that map to a quick draft bacterial chromosome assembly created using Flye (Kolmogorov *et al.* 2019) by default or optionally with Raven (Vaser and Šikić 2021) before conducting hybrid assembly using Unicycler (Wick *et al.* 2017). Plassembler then matches each assembled plasmid contig to the PLSDB (Galata *et al.* 2019) and outputs plasmid copy-number statistics for both long and short-reads. Plassembler can also be used as a fast quality control tool to check that long and short-reads are derived from the same bacterial isolate, which may be particularly useful for users conducting long-read re-sequencing to complete the genomes of isolates previously sequenced with short reads only.

## 2 Materials and methods

**Figure 1.**
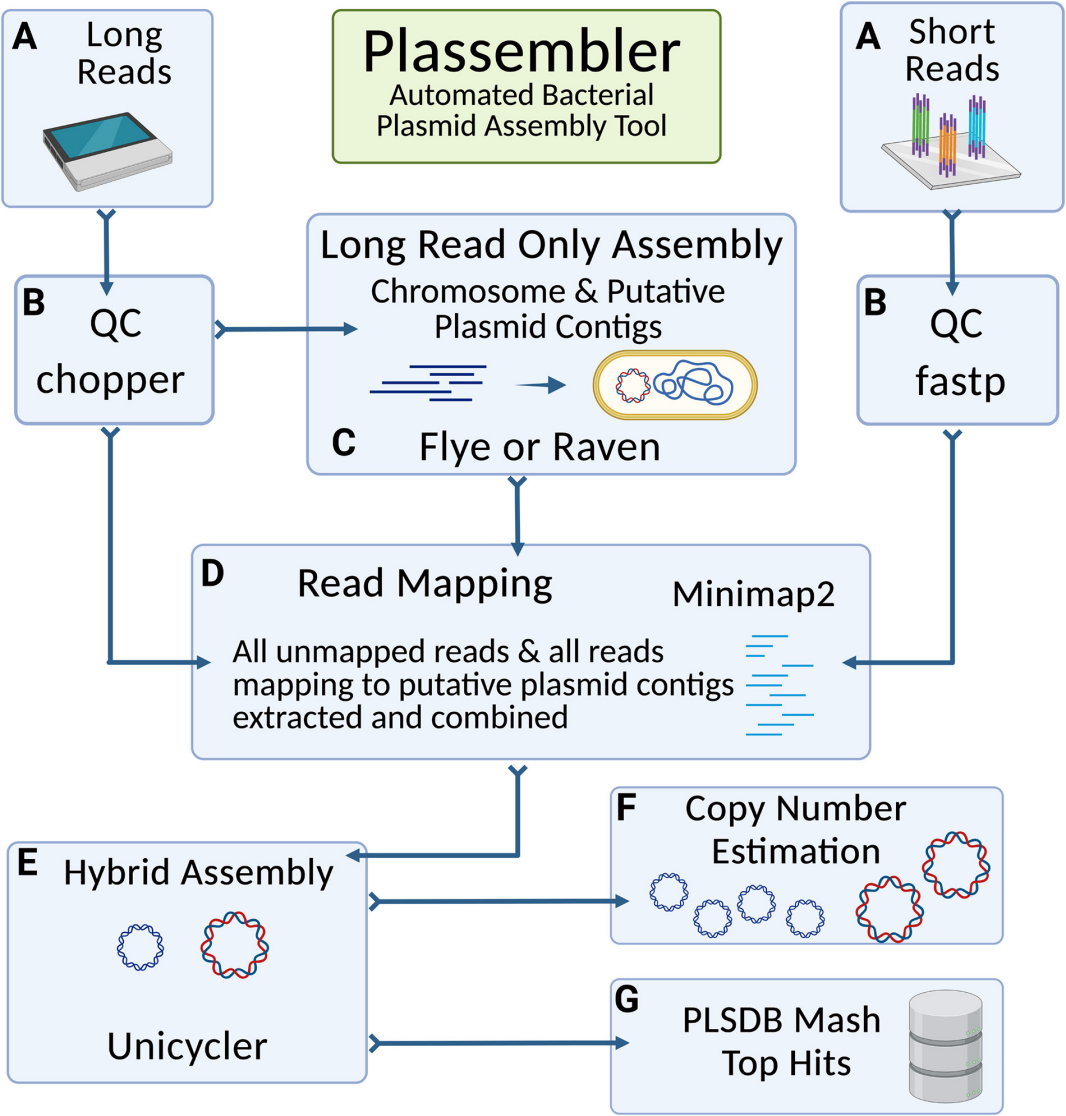
Plassembler workflow. (A) Plassembler requires paired-end short reads and single-end long reads as input. (B) Long reads are filtered using chopper and short reads are filtered and trimmed using fastp. (C) Long-read-first assembly is conducted with Flye by default or optionally with Raven. (D) All long and short reads are mapped to the long-read-first assembly. All reads that are unmapped and all reads that map to putative plasmid contigs are extracted. (E) These reads are then assembled using Unicycler. (F) Plasmid copy number is estimated for each assembled plasmid contig. (G) Each plasmid contig is matched against the PLSDB using mash.

The Plassembler workflow is outlined in Fig. 1.

### 2.1 Input

Plassembler requires hybrid short paired-end and long read single-end FASTQ sequencing reads from the same bacterial isolate, along with a minimum size threshold for classifying chromosomal contigs specified using the ‘-c’ parameter as input (Fig. 1A). Sufficient long-read sequencing depth is required to assemble chromosomal contigs that are larger than the provided threshold (see Section 2.3).

### 2.2 Quality control

Short-read paired-end FASTQs are filtered and trimmed using fastp (Chen *et al.* 2018). Long-read FASTQs are filtered using chopper (De Coster and Rademakers 2023) (Fig. 1B). Plassembler provides the option of filtering the long reads by minimum read length using the ‘-m’ parameter (defaults to 500 bp) and by minimum quality using the ‘-q’ parameter (defaults to a Q-score of 9). Quality control can be skipped using the ‘–skip_qc’ parameter.

### 2.3 Long-read-only assembly

By default, Plassembler uses Flye (Kolmogorov *et al.* 2019) to conduct a long-read-only assembly of the filtered long reads (Fig. 1C). Flye was chosen as the default long-read assembler due to its high chromosome and plasmid recovery, accuracy, and fast runtime (Wick and Holt 2019). If the resulting assembly has at least one contig that is longer than the provided ‘-c’ chromosome length, then all such contigs are denoted as chromosomal and Plassembler continues. Otherwise, Plassembler will exit, asking the user to check the ‘-c’ parameter value input or to increase long-read sequencing depth to ensure a complete chromosome is assembled. If there are additional contigs assembled that are smaller than the provided ‘-c’ chromosome length, Plassembler denotes these as putative plasmid contigs. The ‘-c’ parameter defaults to 1 megabase, allowing for some assembly fragmentation while retaining even large plasmids.

Alternatively, the long read assembler Raven (Vaser and Šikić 2021) can be instead of Flye using the ‘–use_raven’ parameter, which will likely decrease run-time at the potential cost of accuracy (Tables 2 and 3). By default, Plassembler expects Oxford Nanopore Technologies long-reads as input, but can also be used with Pacific Biosciences long-reads using the ‘–pacbio_model’ parameter.

**Table 1.** Plassembler output files.

Output files	Description of file contents
_plasmids.fasta	Final plasmid assembly in FASTA format
_plasmids.gfa	Final plasmid assembly graph in GFA format.
_summary.tsv	Summary file with plasmid length, circularity, estimated copy number statistics for long and short reads (Mean, Standard Deviation, First and Third Quartiles), a column indicating whether the contig had a match in the PLSDB below a mash distance threshold of 0.1 and if so, all available PLSDB information about the top hit.
flye_output or raven_output	Directory holding the output from Flye (default) or Raven (if ‘—use_raven’ is specified).
unicycler_output	Directory holding the output from Unicycler

**Table 2.** Benchmarked time and memory usage.^a^

Threads	Program	Median wall clock time	Min wall clock time	Max wall clock time	Median max memory	Min max memory	Max max memory
1	Plassembler Flye	7012	1926	28 103	3039	2442	5275
1	Plassembler Raven	3893	815	22 106	1764	1307	2464
1	Unicycler	32 619	13 183	66 880	2804	1404	3784
8	Plassembler Flye	1563	531	4852	4842	2274	7712
8	Plassembler Raven	700	132	3051	2579	1587	7891
8	Unicycler	4411	2139	10 003	6568	5610	6826
16	Plassembler Flye	1019	497	2675	5892	3832	15 086
16	Plassembler Raven	430	114	1749	3517	2360	15 195
16	Unicycler	2554	1347	5098	12 967	6509	13 549

a 60× coverage simulated reads from 20 samples. Wall clock time is expressed in seconds (s) and memory is expressed in megabytes (MB).

**Table 3.** Benchmarked time and memory usage.^a^

Threads	Program	Median wall clock time	Min wall clock time	Max wall clock time	Median max memory	Min max memory	Max max memory
1	Plassembler Flye	7063	4559	7865	9921	9116	10 024
1	Plassembler Raven	5918	3430	7028	2623	2214	3039
1	Unicycler	48 325	37 282	58 823	4671	3583	5832
8	Plassembler Flye	1493	1250	1688	10 172	9710	10 897
8	Plassembler Raven	1126	709	1356	6626	2872	8374
8	Unicycler	7500	4509	9659	7535	7003	8128
16	Plassembler Flye	975	779	1163	11 803	10 344	13 467
16	Plassembler Raven	699	397	840	6574	3343	14 784
16	Unicycler	3876	2944	5036	14 041	13 647	14 062

a 60× coverage real reads from six samples from Wick *et al.* (2021a). Wall clock time is expressed in seconds (s) and memory is expressed in megabytes (MB).

### 2.4 Read mapping

Plassembler then maps all long- and short-reads to the long-read-only assembly using Minimap2 (Li 2018) (Fig. 1D). All unmapped reads and all reads that map to putative plasmid contigs are then extracted using SAMtools (Li *et al.* 2009) and combined.

### 2.5 Hybrid assembly and depth estimation

Hybrid assembly is then conducted with Unicycler (Wick *et al.* 2017) to generate final plasmid contigs and assembly graphs (Fig. 1E). Long- and short-read plasmid copy numbers and associated statistics are estimated by mapping all reads to the chromosome and final plasmid assemblies using Minimap2 (Li 2018) and the SAMtools depth function (Li *et al.* 2009, Wick *et al.* 2021a) (Fig. 1F).

### 2.6 PLSDB mash distance calculation

Finally, each assembled plasmid contig is compared to the 34 513 plasmids contained in PLSDB (Galata *et al.* 2019) using mash (Fig. 1G). All matches below the maximum threshold of a mash distance of 0.1 are considered. For each contig, the PLSDB match with the lowest mash distance is kept as the top hit. Contigs that do not have a PLSDB match are denoted as such and are less likely to be true plasmid assemblies, particularly if they are not circular.

## 3 Output

Plassembler’s output files are outlined in Table 1. The primary outputs of Plassembler are a _plasmids.fasta file and a _plasmids.gfa file. The _plasmids.fasta file is taken from the output of Unicycler and contains the final plasmid assemblies in FASTA format. This is suitable for downstream analysis using tools such as MOB-suite (Robertson and Nash 2018) and mge-cluster (Arredondo-Alonso *et al.* 2022). In addition, a ‘_plasmids.gfa’ file is generated containing the Unicycler assembly graphs that can be visualized using tools like Bandage (Wick *et al.* 2015). In addition, plassembler provides a ‘_summary.tsv’ file. This file includes each plasmid’s length, estimated mean, first quartile, third quartile and standard deviation of each plasmid’s short-read and long-read depths, a column indicating whether each plasmid contig is circular and a column indicating whether the contig has a match in PLSDB under the maximum mash distance threshold of 0.1. If there is a hit, the ‘_summary.tsv’ file will also contain all available PLSDB information about the top hit.

## 4 Benchmarking

Benchmarking, implemented using a reproducible Snakemake pipeline (Mölder *et al.* 2021) powered by Snaketool (Roach *et al.* 2022), was conducted on an Intel^®^ Core™ i7-10700K CPU @ 3.80 GHz on a machine running Ubuntu 20.04.6 LTS. To test the performance of Plassembler, we used simulated reads from 20 isolate assemblies from four different datasets. These consisted of:

1. six isolate assemblies and read sets from Wick *et al.* (2021a) (available at https://bridges.monash.edu/articles/dataset/Small_plasmid_Nanopore_data/13543754) assembled with Trycycler and manually curated in that study.
2. one *Staphylococcus aureus* isolate assembly (C222 SAMN32360844 in BioProject PRJNA914892 in the NCBI BioProject database, specifically SRR22859710 for ONT long reads and SRR22859843 for short reads) previously sequenced by us as an example of a bacterial isolate with one small plasmid (Houtak *et al.* 2023).
3. twelve *Enterobacteriaceae* isolates with from De Maio *et al.* (2019) that contained at least one plasmid with accessible sample numbers in the European Nucleotide Archive. We used the subsampled Oxford Nanopore Technologies Unicycler assemblies as this was the best performing method in that study.
4. one *Klebsiella pneumoniae* strain CAV1217 assembly as an example of a challenging isolate testing the ability of Plassembler to process multi-mapped reads. It has a 16 kb mobile genetic element present in both the chromosome and on a 44 kb plasmid (Mathers *et al.* 2017).

We used Badread v0.3.0 (Wick 2019) and InSilicoSeq v1.5.4 (Gourlé *et al.* 2019) to generate simulated readsets from all ground truth assemblies. Long reads were simulated with the Nanopore 2020 error model, while short reads were simulated with the ‘novaseq’ error model. Both long and short read sets were simulated to a genome coverage of 60×.

In addition to the simulated readsets, we tested the performance of Plassembler on real reads from the six isolates from (Wick *et al.* 2021a). Because these genomes were assembled using a highly accurate and independent approach to that used by Plassembler [Trycycler (Wick *et al.* 2021b) with manual curation], we considered that these assemblies could also be used as ground truth for testing the accuracy of Plassembler on the corresponding real readsets. Wick, Judd, Wyers, *et al.* have made all the details of their methodology available at https://github.com/rrwick/Small-plasmid-Nanopore/blob/main/method.md. These isolates were sequenced in two technical replicates with two long-read sequencing methods. For our study, reads for both technical replicates and both sequencing chemistries were combined and subsampled to a depth of 60× using rasusa v0.7.0 (Hall 2022).

To assess computational performance, Plassembler v1.1.0 (with Unicycler v0.5.0, SPAdes v3.15.5, Flye v2.9.2 and Raven v1.8.1) using both Flye and Raven was compared against Unicycler v0.5.0 (with SPAdes v3.15.5) in terms of speed and accuracy, using 1, 8, and 16 threads. To assess accuracy, Plassembler assemblies for the 20 simulated read sets (with a verifiable ground truth) and the six Wick *et al.* (2021a) real read sets (with an independent manually curated ground truth), were compared to the ground truth assemblies using QUAST to assess genome fraction, mismatches per 100 kb and indels per 100 kb (Gurevich *et al.* 2013).

## 5 Results

Plassembler was faster than Unicycler for every sample for the 20 simulated isolates and six real read sets samples for all thread counts, yielding a 3- to 10-fold speed improvement (Tables 2 and 3) depending on the sample, thread count, and long-read assembler used. The decrease in wall-clock runtime was largest single-threaded. Plassembler and Unicycler both had comparable maximum memory usage.

Plassembler was more accurate than Unicycler overall, recovering a higher average QUAST genome fraction than Unicycler against the simulated ground truth (Table 4). For the simulated reads, Plassembler missed fewer plasmids (one versus seven for Unicycler), but had a higher number of fragmented assemblies (four for Plassembler with Flye, five for Plassembler with Raven versus one for Unicycler). Unicycler also had one misassembly, while Plassembler did not have any. Rates of indels and mismatches were comparable and low for all three assembly methods.

**Table 4.** Benchmarked accuracy.^a^

Program	Complete plasmids	Missed plasmids	Incomplete plasmids	Misassemblies	Genome fraction	Indels	Mismatches
Plassembler Flye	69	1	4	0	99.66 (mean), 99.94 (median), (97.68, 100)	0 (0, 1.37)	0.91 (0, 11.12)
Plassembler Raven	68	1	5	0	99.47 (mean), 99.96 (median), (96.59, 100)	0 (0, 1.41)	0.91 (0, 11.12)
Unicycler	65	7	1	1	93.81 (mean), 99.88 (median), (0, 100)	0 (0, 1.54)	0.88 (0, 7.28)

a 60× coverage simulated reads from 20 samples. Median values indicated for Indels and Mismatches. Minimums and maximums in brackets. Indels and mismatches calculated per 100 kb. Results from eight threads.

The difference in genome fraction is explained by Plassembler’s ability to recover small plasmids under 10 kb. In the simulated read sets Plassembler was able to recover small plasmids in *Staphylococcus aureus* C222 (2473 bp), *Citrobacter koseri* MINF 9D (9294 bp), *Klebsiella oxytoca* MSB1 2C (4574 bp), *Klebsiella variicola* INF345 (5783 bp), *Enterobacter cloacae* RBHSTW-00059 (2495 bp), and *Klebsiella pneumonaie* RBHSTW-00128 (3980 bp) that were missed by Unicycler (Supplementary Table S1).

For the real read sets, Plassembler, and Unicycler had identical genome fractions and low indel and mismatch rates (Table 5). Similar to the simulated dataset, Plassembler recovered two additional small plasmids missed by Unicycler (Table 5 and Supplementary Table S6) of lengths 1934 bp (*K.variicola* INF345) and 10 697 bp (*K.oxytoca* MSB1 2C). The 10 697 bp plasmid recovered in *K.oxytoca* MSB1 2C was not recovered using the long-read first assembly method by Wick *et al.* (2021a). Annotation with Bakta v1.7.0 (Schwengers *et al.* 2021) revealed that this plasmid contains a Type III toxin-antitoxin system and other plasmid replication genes (Supplementary Table S10).

**Table 5.** Benchmarked accuracy.^a^

Program	Complete plasmids	Additional recovered plasmids	Missed plasmids	Incomplete plasmids	Misassemblies	Genome fraction	Indels	Mismatches
Plassembler Flye	20	2	2	1	0	97.02 (mean), 100 (median), (82.14, 100)	0.46 (0, 1.67)	0.39 (0, 6.55)
Plassembler Raven	20	2	2	1	0	97.02 (mean), 100 (median), (82.14, 100)	0.64 (0, 1.67)	0.78 (0, 7.28)
Unicycler	20	0	2	1	0	97.02 (mean), 100 (median), (82.14, 100)	0.18 (0, 1.67)	0.18 (0, 2.65)

a Real 60× subsampled reads from six samples from Wick *et al.* (2021a) Median values indicated for indels and mismatches. Minimums and maximums in brackets. Indels and mismatches calculated per 100 kb. Results from eight threads.

Plassembler with Raven was consistently faster than Plassembler with Flye (Tables 2 and 3). However, Plassembler with Raven had more fragmented assemblies in the simulated dataset (Table 4), due to worse performance of Raven in recovering draft assemblies of some plasmids compared to Flye (Wick and Holt 2019) (Supplementary Table S1).

## 6 Discussion

It has previously been shown that subsampling hybrid sequencing reads sets leads to increased plasmid recovery (De Maio *et al.* 2019). Plassembler’s removal of chromosomal reads before short-read first assembly has similar benefits in terms of small plasmid recovery, as small plasmid reads constitute a larger proportion of the overall read set.

Flye and especially Raven assemblies commonly miss small plasmids (Supplementary Table S1), emphasizing that a long-read first-assembly approach is inappropriate for recovering small plasmids, as reported in other studies (Wick *et al.* 2023, Johnson *et al.* 2023).

Long read first assemblies with Flye (run as a part of Plassembler) in the real read datasets multiplicated many small plasmids (Supplementary Table S6). Multiplication was also present, though less common, in the simulated datasets (Supplementary Table S2). As reported previously (Wick and Holt 2019, Johnson *et al.* 2023), this indicates that multiplication in long read only plasmid assemblies may either reflect assembly errors or true plasmid multimerization (Crozat *et al.* 2014), but it is difficult to distinguish between the two.

### 6.1 Other use cases and features

Plassembler can be used to recover small plasmids from bacteria with multiple chromosomes, megaplasmids, or chromids. Plassembler will treat all long-read assembled contigs larger than the provided ‘-c’ parameter as chromosomal. As an example, Plassembler v1.1.0 was used to recover plasmids from *Vibrio campellii* DS40M4, has two chromosomes of sizes 3.33 and 1.88 Mb and a 77 353 bp plasmid (Colston *et al.* 2019). Illumina and ONT sequencing reads for *V.campellii* were downloaded using fastq-dl (Petit III and Hall) https://github.com/rpetit3/fastq-dl. Plassembler recovered the known 77 353 bp plasmid and an additional 5386 bp replicon (Supplementary Table S9), which blastn (Sayers *et al.* 2022) revealed was Enterobacteria phage phiX174, which is commonly used as a positive control in short-read sequencing runs and likely reflects contamination in this sample.

Plassembler can also be used as a fast quality control tool to detect differences between long and short read sets even from closely related isolates. From readsets of different isolates from the same species, Plassembler will extract all short- and long-reads that are unmapped to the long-read-only assembly. The hybrid assembly of these reads will then contain sections of chromosomal sequence that are present in the short-read set genome but not the long read set genome. These will be represented as noncircular contigs in the Plassembler output, likely without a PLSDB mash hit. Therefore, if five or more such contigs are assembled, Plassembler will warn the user that their long- and short- read sets may not match. Examples of Plassembler output where read sets from two closely related but distinct *S.aureus* isolates (same sequence type), and also two more distantly related *S.aureus* isolates (different sequence types) can be found in Supplementary Tables S11 and S12 (Enright *et al.* 2000, Houtak *et al.* 2023).

In addition, users with existing plasmid and chromosome assemblies who wish to estimate long and short read plasmid copy numbers and match each plasmid to the PLSDB can use Plassembler. This is enabled using ‘plassembler assembled’, along with specifying the assembled chromosome using the ‘–input_chromosome’ and the plasmids using ‘–input_plasmids’.

Plassembler can also be used to assemble other small extrachromosomal replicons in hybrid sequencing data, such as bacteriophages (Shen and Millard 2021) or phage-plasmids (Pfeifer *et al.* 2022), assuming they have not integrated into the chromosome. An example is the 5386bp Enterobacteria phage phiX174 Plassembler recovered from Colston *et al.* (2019) mentioned above.

### 6.2 Limitations

Plassembler is nondeterministic between threadcounts, which is caused by long-read assembler nondeterminism (Supplementary Table S1). This leads to different read sets being recovered in Plassembler’s mapping process, which occasionally produces differing plasmid assemblies. With Flye, nondeterminism also persisted even where the ‘–deterministic’ parameter was used (Supplementary Table S5).

Plassembler requires sufficient long-read depth such that Flye or Raven can assemble complete chromosome-sized contigs. Plassembler therefore cannot be used with isolates with extremely low read depth. Unicycler should be used in this scenario.

The known linear plasmid in *K.variicola* INF345 reported by Wick *et al.* (2021a) was incorrectly assembled by both Plassembler and Unicycler in simulated and real read sets, due to a terminal inverted repeat that is characteristic of linear plasmids (Hawkey *et al.* 2022) (Supplementary Table S1). It is likely that linear plasmids are better assembled using a long-read first approach.

Another possible limitation of Plassembler is with small plasmids that contain a mobile genetic element (MGE) shared with the chromosome. If the long-read assembler fails to assemble the small plasmid, then the Plassembler assembly will be incomplete. This is because reads that map to the MGE on the plasmid will neither be unmapped to the chromosome nor map to plasmid contigs in the Plassembler mapping process. Based on our benchmarking, this is unlikely to be an issue for plasmids larger than 10 kb, as the long read assembler is likely to recover them. Plassember was able to accurately recover the 44 kb plasmid harbouring a 16 kb mobile genetic element (MGE) shared by both the chromosome and plasmid for *K. pneumoniae* CAV 1217 (Supplementary Table S1).

## 7 Conclusion

Plassembler assembles bacterial plasmids from hybrid sequencing datasets faster and more accurately than existing approaches. It recovers more small plasmids that other assemblers miss and can be easily combined with long-read-first chromosomal assembly workflows to generate accurate bacterial genome assemblies.

## Supplementary Material

btad409_Supplementary_Data

## References

[btad409-B1] Arredondo-Alonso S , Gladstone RA, Pöntinen AK et al Consistent typing of plasmids with the mge-cluster pipeline. *bioRxiv* 2022, 10.1101/2022.12.16.520696.

[btad409-B2] Bankevich A , Nurk S, Antipov D et al SPAdes: a new genome assembly algorithm and its applications to single-cell sequencing. J Comput Biol 2012;19:455–77. 22506599 10.1089/cmb.2012.0021 PMC3342519

[btad409-B3] Barry KE , Wailan AM, Sheppard AE et al Don’t overlook the little guy: an evaluation of the frequency of small plasmids co-conjugating with larger carbapenemase gene containing plasmids. Plasmid 2019;103:1–8. 30928702 10.1016/j.plasmid.2019.03.005

[btad409-B4] Chen S , Zhou Y, Chen Y et al fastp: an ultra-fast all-in-one FASTQ preprocessor. Bioinformatics 2018;34:i884–90. 30423086 10.1093/bioinformatics/bty560 PMC6129281

[btad409-B5] Colston SM , Hervey WJ, Horne WC et al Complete genome sequence of *Vibrio campbellii* DS40M4. Microbiol Resour Announc 2019;8:e01187-18. 30701232 10.1128/MRA.01187-18 PMC6346181

[btad409-B6] Crozat E , Fournes F, Cornet F et al Resolution of multimeric forms of circular plasmids and chromosomes. Microbiol Spectr 2014;2:37. 10.1128/microbiolspec.PLAS-0025-2014 26104344

[btad409-B7] De Coster W , Rademakers R. NanoPack2: population-scale evaluation of long-read sequencing data. Bioinformatics 2023;39:btad311. 37171891 10.1093/bioinformatics/btad311 PMC10196664

[btad409-B8] De Maio N , Shaw LP, Hubbard A et al Comparison of long-read sequencing technologies in the hybrid assembly of complex bacterial genomes. Microb Genomics 2019;5:e000294. 10.1099/mgen.0.000294 PMC6807382 31483244

[btad409-B9] Enright MC , Day NP, Davies CE et al Multilocus sequence typing for characterization of methicillin-resistant and methicillin-susceptible clones of *Staphylococcus aureus*. J Clin Microbiol 2000;38:1008–15. 10698988 10.1128/jcm.38.3.1008-1015.2000 PMC86325

[btad409-B10] Galata V , Fehlmann T, Backes C et al PLSDB: a resource of complete bacterial plasmids. Nucleic Acids Res 2019;47:D195–202. 30380090 10.1093/nar/gky1050 PMC6323999

[btad409-B11] Gourlé H , Karlsson-Lindsjö O, Hayer J et al Simulating Illumina metagenomic data with InSilicoSeq. Bioinformatics 2019;35:521–2. 30016412 10.1093/bioinformatics/bty630 PMC6361232

[btad409-B12] Gurevich A , Saveliev V, Vyahhi N et al QUAST: quality assessment tool for genome assemblies. Bioinformatics 2013;29:1072–5. 23422339 10.1093/bioinformatics/btt086 PMC3624806

[btad409-B13] Hall MB. Rasusa: randomly subsample sequencing reads to a specified coverage. JOSS 2022;7:3941.

[btad409-B14] Hawkey J , Cottingham H, Vyahhi N et al Linear plasmids in Klebsiella and other Enterobacteriaceae. Microb Genomics 2022;8:000807. 10.1099/mgen.0.000807 PMC9453081 35416146

[btad409-B15] Houtak G , Bouras G, Nepal R et al The intra-host evolutionary landscape and pathoadaptation of persistent *Staphylococcus aureus* in chronic rhinosinusitis. bioRxiv, 10.1101/2023.03.28.534496, 2023, preprint: not peer reviewed. PMC10711304 38010322

[btad409-B16] Johnson J , Soehnlen M, Blankenship HM et al Long read genome assemblers struggle with small plasmids. Microb Genomics 2023;9:001024. 10.1099/mgen.0.001024 PMC10272865 37224062

[btad409-B17] Kolmogorov M , Yuan J, Lin Y et al Assembly of long, error-prone reads using repeat graphs. Nat Biotechnol 2019;37:540–6. 30936562 10.1038/s41587-019-0072-8

[btad409-B18] Li H. Minimap2: pairwise alignment for nucleotide sequences. Bioinformatics 2018;34:3094–100. 29750242 10.1093/bioinformatics/bty191 PMC6137996

[btad409-B19] Li H , Handsaker B, Wysoker A, 1000 Genome Project Data Processing Subgroup et al The sequence alignment/map format and SAMtools. Bioinformatics 2009;25:2078–9. 19505943 10.1093/bioinformatics/btp352 PMC2723002

[btad409-B20] Mathers AJ , Stoesser N, Chai W et al Chromosomal integration of the Klebsiella pneumoniae Carbapenemase Gene, blaKPC, in Klebsiella species is elusive but not rare. Antimicrob Agents Chemother 2017;61:e01823-16. 10.1128/AAC.01823-16 PMC5328509 28031204

[btad409-B151] Mölder F , Jablonski KP, Letcher B et al Sustainable data analysis with Snakemake. F1000Research 2021. 10.12688/f1000research.29032.1 PMC8114187 34035898

[btad409-B21] Murigneux V , Roberts LW, Forde BM et al MicroPIPE: validating an end-to-end workflow for high-quality complete bacterial genome construction. BMC Genomics 2021;22:474. 34172000 10.1186/s12864-021-07767-z PMC8235852

[btad409-B22] Petit RA III , Hall MB. fastq-dl: efficiently download FASTQ files from SRA or ENA repositories. https://github.com/rpetit3/fastq-dl.

[btad409-B23] Pfeifer E , Bonnin RA, Rocha EPC et al Phage-plasmids spread antibiotic resistance genes through infection and lysogenic conversion. mBio 2022;13:e01851-22. 36154183 10.1128/mbio.01851-22 PMC9600943

[btad409-B24] Roach MJ , Pierce-Ward NT, Suchecki R et al Ten simple rules and a template for creating workflows-as-applications. PLoS Comput Biol 2022;18:e1010705. 36520686 10.1371/journal.pcbi.1010705 PMC9754251

[btad409-B25] Robertson J , Nash JHE. MOB-suite: software tools for clustering, reconstruction and typing of plasmids from draft assemblies. Microb Genomics 2018;4:e000206. 10.1099/mgen.0.000206 PMC6159552 30052170

[btad409-B26] Sayers EW , Bolton EE, Brister JR et al Database resources of the national center for biotechnology information. Nucleic Acids Res 2022;50:D20–6. 34850941 10.1093/nar/gkab1112 PMC8728269

[btad409-B27] Schwengers O , Jelonek L, Dieckmann MA et al Bakta: rapid and standardized annotation of bacterial genomes via alignment-free sequence identification. Microb Genomics 2021;7:000685. 10.1099/mgen.0.000685 PMC8743544 34739369

[btad409-B28] Shen A , Millard A. Phage genome annotation: where to begin and end. Phage (New Rochelle) 2021;2:183–93. 36159890 10.1089/phage.2021.0015 PMC9041514

[btad409-B29] Vaser R , Šikić M. Time- and memory-efficient genome assembly with Raven. Nat Comput Sci 2021;1:332–6. 10.1038/s43588-021-00073-4 38217213

[btad409-B30] Wick RR. Badread: simulation of error-prone long reads. JOSS 2019;4:1316.

[btad409-B31] Wick RR , Judd LM, Gorrie CL et al Unicycler: resolving bacterial genome assemblies from short and long sequencing reads. PLoS Comput Biol 2017;13:e1005595. 28594827 10.1371/journal.pcbi.1005595 PMC5481147

[btad409-B32] Wick RR , Holt KE. Benchmarking of long-read assemblers for prokaryote whole genome sequencing. F1000Res 2019;8:2138. 31984131 10.12688/f1000research.21782.1 PMC6966772

[btad409-B33] Wick RR , Judd LM, Wyres KL et al Recovery of small plasmid sequences via Oxford Nanopore sequencing. Microb Genomics2021a;7:000631. 10.1099/mgen.0.000631 PMC8549360 34431763

[btad409-B34] Wick RR , Judd LM, Cerdeira LT et al Trycycler: consensus long-read assemblies for bacterial genomes. Genome Biol2021b;22:266. 34521459 10.1186/s13059-021-02483-z PMC8442456

[btad409-B35] Wick RR , Judd LM, Holt KE et al Assembling the perfect bacterial genome using Oxford Nanopore and Illumina sequencing. PLoS Comput Biol 2023;19:e1010905. 36862631 10.1371/journal.pcbi.1010905 PMC9980784

[btad409-B152] Wick RR , Schultz MB, Zobel J et al Bandage: interactive visualization of de novo genome assemblies. Bioinformatics 2015;31:3350–2. 26099265 10.1093/bioinformatics/btv383 PMC4595904

