# Supplementary material for: Plassembler: an automated bacterial plasmid assembly tool
Source: Bioinformatics. 2023 Jun 27;39(7):btad409. doi: 10.1093/bioinformatics/btad409 (PMC10326302; doi:10.1093/bioinformatics/btad409)
Supplement: btad409_Supplementary_Data [file btad409_supplementary_data.zip › Supplementary Tables Legend.docx]

# Supplementary Data

Supplementary Table 1: Table showing the assembled plasmid lengths for each simulated read set assembly in benchmarking, along with counts of missed or fragmented plasmids.

Supplementary Table 2: Table showing duplications or triplications in simulated read set Flye assemblies, derived from the Plassembler output.

Supplementary Table 3: Table containing the QUAST output for each simulated read set assembly in benchmarking, compared against the ground truth assembly.

Supplementary Table 4: Table containing the benchmarking statistics for each simulated read set assembly.

Supplementary Table 5: Table showing the assembled contig lengths using Plassembler with and without ‘--deterministic’ parameter indicated in Flye.

Supplementary Table 6: Table showing the assembled plasmid lengths for each real read set assembly in benchmarking, along with counts of missed or fragmented plasmids, and also additional plasmids recovered by Plassembler no found in the ground truth.

Supplementary Table 7: Table containing the benchmarking statistics for each real read set assembly.

Supplementary Table 8: Table containing the QUAST output for each real read set assembly in benchmarking, compared against the ground truth assembly.

Supplementary Table 9: Plassembler v1.1.0 output table for *Vibrio campellii* DS40M4 sequenced in Colston, *et al*. 2019.

Supplementary Table 10: TSV file containing the Bakta v1.7.0 annotation of the 10697 bp plasmid Plassembler v1.1.0 recovered in the real read sets of *K. oxytoca* MSB1 2C from Wick, Judd, Wyres, *et al.*, 2021.

Supplementary Table 11: Plassembler v1.1.0 output table for *S. aureus* SAMN32360844 long read set assembled with SAMN32360859 short read set from Houtak, *et al*. 2023 (both Sequence Type 22).

Supplementary Table 12: Plassembler v1.1.0 output table for *S. aureus* SAMN32360844 long read set assembled with SAMN32360859 short read set from Houtak, *et al*. 2023 (Sequence Type 22 and Sequence Type 30).
